# HIV-1 Vpr Reprograms CLR4^DCAF1^ E3 Ubiquitin Ligase to Antagonize Exonuclease 1-Mediated Restriction of HIV-1 Infection

**DOI:** 10.1128/mBio.01732-18

**Published:** 2018-10-23

**Authors:** Junpeng Yan, Ming-Chieh Shun, Caili Hao, Yi Zhang, Juan Qian, Kasia Hrecka, Maria DeLucia, Christina Monnie, Jinwoo Ahn, Jacek Skowronski

**Affiliations:** a Department of Molecular Biology and Microbiology, Case Western Reserve School of Medicine, Cleveland, Ohio, USA; b Department of Structural Biology, University of Pittsburgh School of Medicine, Pittsburgh, Pennsylvania, USA; University of Pittsburgh School of Medicine

**Keywords:** DNA repair, human immunodeficiency virus, innate immunity, protein degradation, ubiquitination

## Abstract

Viral accessory proteins hijack host cell E3 ubiquitin ligases to antagonize innate/intrinsic defenses and thereby provide a more permissive environment for virus replication. Human immunodeficiency virus type 1 (HIV-1) accessory protein Vpr reprograms CRL4^DCAF1^ E3 to antagonize select postreplication DNA repair enzymes, but the significance and role of these Vpr interactions are poorly understood. To gain additional insights, we performed a focused screen for substrates of CRL4^DCAF1^ E3 reprogrammed by HIV-1 Vpr among known postreplication DNA repair proteins and identified exonuclease 1 (Exo1) as a novel direct HIV-1 Vpr target. We show that HIV-1 Vpr recruits Exo1 to the CRL4^DCAF1^ E3 complex for ubiquitination and subsequent proteasome-dependent degradation and that Exo1 levels are depleted in HIV-1-infected cells in a Vpr-dependent manner. We also show that Exo1 inhibits HIV-1 replication in T cells. Notably, the antagonism of Exo1 is a conserved function of main group HIV-1 and its ancestor Vpr proteins in the simian immunodeficiency virus from chimpanzee (SIVcpz) lineage, further underscoring the relevance of our findings. Overall, our studies (i) reveal that HIV-1 Vpr extensively remodels the cellular postreplication DNA repair machinery by impinging on multiple repair pathways, (ii) support a model in which Vpr promotes HIV-1 replication by antagonizing select DNA repair enzymes, and (iii) highlight the importance of a new class of restrictions placed on HIV-1 replication in T cells by the cellular DNA repair machinery.

## Abstract (precis)

HIV-1 polymerase reverse transcribes the viral RNA genome into imperfectly double-stranded proviral DNA, containing gaps and flaps, for integration into the host cell chromosome. HIV-1 reverse transcripts share characteristics with cellular DNA replication intermediates and are thought to be converted into fully double-stranded DNA by cellular postreplication DNA repair enzymes. Therefore, the finding that the HIV-1 accessory protein Vpr antagonizes select postreplication DNA repair enzymes that can process HIV-1 reverse transcripts has been surprising. Here, we show that one such Vpr-antagonized enzyme, exonuclease 1, inhibits HIV-1 replication in T cells. We identify exonuclease 1 as a member of a new class of HIV-1 restriction factors in T cells and propose that certain modes of DNA “repair” inhibit HIV-1 infection.

## INTRODUCTION

Viral accessory proteins counteract innate antiviral defenses of the host cell to provide a more permissive environment for viral replication. Identification of biochemical targets of accessory proteins has revealed key mechanisms that restrict virus replication in the host (1–3).

The human immunodeficiency virus type 1 (HIV-1) accessory proteins Vpr, Vif, and Vpu reprogram cellular E3 ubiquitin ligases to direct key host cell proteins that restrict HIV infection for proteasome-dependent degradation. In particular, Vif usurps a cellular RING ubiquitin ligase assembled on a Cullin 5 scaffold (CRL5) to antagonize APOBEC3 family cytidine deaminases, which are capable of inducing lethal G-to-A hypermutation in viral cDNA (4). Vpu hijacks a CRL1 E3 ubiquitin ligase complex to antagonize the CD4 receptor (5), thereby preventing superinfection, and to deplete select proteins that are involved in interferon-inducible antiviral pathways (6). Overall, how Vif and Vpu exploit cellular E3 enzymes to facilitate HIV-1 infection is relatively well understood.

In contrast to those of Vif and Vpu, the biochemical mechanisms mediating Vpr function in HIV-1 infection are less clear. Vpr hijacks a multisubunit CRL4^DCAF1^ E3 ubiquitin ligase complex comprising the Cullin 4 (Cul4) scaffold, DDB1 linker protein, DDA1 core subunit (7), and DCAF1 substrate receptor, to which Vpr binds (8–10). Several tentative target proteins of the Vpr-CRL4^DCAF1^ E3 were recently identified, four of which are bona fide Vpr-recruited substrates (11–13). The latter includes proteins identified by unbiased screens and have well-established roles in postreplication DNA repair, such as uracil-DNA glycosylase 2 (UNG2) (14), MUS81 (15), and helicase like transcription factor (HLTF) (16, 17), or in epigenetic control of gene expression, such as ten eleven translocation methylcytosine dioxygenase 2 (Tet2) (11), which, notably, is also involved in DNA damage response and repair (18). More specifically, UNG2, a nuclear isoform of UNG, excises uracil from DNA that results from misincorporation of dUMP by DNA polymerase or from cytosine deamination, thus initiating base excision repair (19). dUTP concentrations are relatively high in macrophages, and uracil incorporation may flag viral DNA for restrictive processing by cellular DNA repair enzymes in these cells (20, 21). MUS81 and HLTF process branched DNA structures, such as those found at stalled replication forks. The MUS81-EME1 (or -EME2) heterodimer is a DNA structure-selective endonuclease involved in homologous recombination and replication fork processing and repair (22, 23). HLTF is a DNA helicase which catalyzes strand reversal at damaged replication forks (24).

The recently revealed narrow clustering of biochemically validated, Vpr-antagonized targets within postreplication DNA repair machinery is intriguing, as it suggests that DNA repair enzymes might impinge on HIV-1 replication to a much larger extent than currently appreciated. Noteworthy, many of the previously reported effects of HIV-1 Vpr on HIV-1 replication, including the modulation of the levels of reverse transcription intermediates, the facilitation of nuclear import of HIV-1 preintegration complex, the modulation of HIV-1 mutation rate in macrophages (25), and the modulation of innate sensing of HIV-1 infection (26), might be indirect consequences/manifestations of the counteraction of DNA repair processing of HIV-1 DNA by Vpr.

Vpr also elicits hallmarks of cellular responses to damaged DNA, probably by activating the DNA damage checkpoint controlled by ataxia telangiectasia and Rad3-related protein (ATR) kinase (27). Significantly, Vpr interaction with CRL4^DCAF1^ E3 has been genetically linked to its effects on DNA damage checkpoint activation (7, 28, 29) and innate sensing of HIV-1 infection via the cytosolic DNA sensor cyclic GMP-AMP synthase (cGAS) (26). Yet, the so-far-identified targets of Vpr antagonism do not fully explain the reported cellular and virologic effects of Vpr expression, suggesting that additional substrates of the Vpr-CRL4^DCAF1^ E3 may exist.

Here, we carried out a focused screen aiming to more fully define the spectrum of postreplication DNA repair proteins antagonized by HIV-1 Vpr via CRL4^DCAF1^ E3. Our studies identify exonuclease 1 (Exo1) as a new target of HIV-1 Vpr antagonism. Exo1 is a multifunctional nuclease involved in multiple DNA repair pathways, including nucleotide excision repair (NER), resection of DNA ends during single- and double-stranded break repair, and homologous recombination processes (30). We show that (i) Exo1 levels are depleted in HIV-1-infected CD4^+^ T cells in a Vpr-dependent manner, (ii) Vpr loads Exo1 onto the CRL4^DCAF1^ E3 complex *in vivo* and *in vitro*, and (iii) the ability to antagonize Exo1 is a conserved function of Vpr from the main groups of HIV-1 strains and their ancestor simian immunodeficiency viruses from chimpanzees (SIVcpzs). Together, our studies identify a key postreplication DNA repair enzyme as a target of HIV-1 Vpr, reinforce the finding that Vpr usurps CRL4^DCAF1^ to antagonize major DNA repair mechanisms, provide indirect support of the model in which the cellular postreplication DNA machinery restricts HIV-1 replication, and provide a stage for systematic dissection of the roles of Vpr-targeted DNA repair enzymes in the HIV-1 replication cycle.

## RESULTS

### Targeted screen for HIV-1 Vpr-CRL^DCAF1^ E3 substrate proteins involved in postreplication DNA repair identifies Exo1 as a Vpr target.

We set up a targeted screen aiming to identify novel proteins that are involved in postreplication DNA repair and are directed for proteasome-mediated degradation via HIV-1 Vpr bound to CLR4^DCAF1^ E3. To this end, we searched for proteins that were depleted in U2OS cells inducibly expressing a well-characterized HIV-1 NL4-3 Vpr protein (U2OS-iH1vpr). Levels of approximately 30 proteins, including DNA damage sensors, nucleases, and helicases involved in major postreplication DNA repair pathways, such as base and nucleotide excision repair, DNA strand break processing, and recombination, were characterized. More specifically, lysates prepared from U2OS-iH1vpr cells induced with doxycycline to express Vpr or prepared from noninduced control U2OS-iH1vpr and parental U2OS cell populations were characterized by immunoblotting. As shown in Fig. S1 in the supplemental material, these experiments revealed that exonuclease 1 (Exo1) levels are specifically depleted in U2OS-iH1vpr cells following induction of Vpr expression but not in control cell populations that did not express Vpr, as were the levels of UNG2, as expected. Thus, our targeted screen tentatively identified Exo1 as a novel target of the CRL4^DCAF1^ E3 subverted by HIV-1 Vpr.

10.1128/mBio.01732-18.1 FIG S1 Targeted screen for HIV-1 Vpr-CRL^DCAF1^ E3 substrate proteins involved with postreplication DNA repair. Lysates of U2OS_iH1vpr (iH1vpr) and control U2OS cells (mock), cultured in the absence or presence of doxycycline (100 ng/ml) for 48 hours, were immunoblotted for the indicated proteins. Download FIG S1, TIF file, 0.5 MB. Copyright © 2018 Yan et al. 2018 Yan et al. This content is distributed under the terms of the Creative Commons Attribution 4.0 International license.

### HIV-1 infection depletes Exo1 levels in human CD4^+^ T cells in a Vpr-dependent manner.

**FIG 1:**
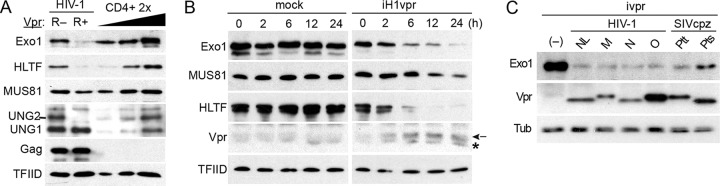
HIV-1 Vpr depletes Exo1 levels in CD4^+^ T cells. (A) HIV-1 infection depletes Exo1 in primary CD4^+^ T cells in a Vpr-dependent manner. Human peripheral blood CD4^+^ T cells were activated with α-CD3/α-CD28 beads and 2 days later challenged with HIV-1 NL4-3.GFP.*R*+ (or R–) single-cycle viruses expressing GFP marker protein. Exo1, HLTF, MUS81, and UNG2/UNG1 levels in extracts from productively infected cells, isolated by cell sorting for GFP fluorescence, were characterized by immunoblotting. Twofold serial dilutions of control CD4^+^ T cell extracts provided quantification standards. Levels of HIV-1 Gag were also characterized to confirm similar infections with the R– and *R*+ viruses. TFIID provided a loading control. (B) Exo1 is rapidly depleted in cells expressing HIV-1 Vpr. U2OS-iH1vpr (iH1vpr) and control U2OS (mock) cells were cultured in the presence of doxycycline (100 ng/ml). Extracts prepared from the cells at the indicated times postaddition of doxycycline were analyzed by immunoblotting for Exo1 and other postreplication DNA repair proteins known to be antagonized by HIV-1 Vpr. The arrow indicates the position of the Vpr band, and the asterisk indicates a nonspecifically cross-reacting background band. (C) Exo1 is a conserved target of main group HIV-1 and SIVcpz Vpr proteins. Levels of Exo1, Vpr, and a tubulin loading control in extracts from U2OS cells induced with doxycycline (100 ng/ml) to express the HIV-1 M, N, or O main group consensus Vpr proteins or SIVcpz P. troglodytes *troglodytes* (Ptt) or SIVcpz P. troglodytes *schweinfurthii* (Pts) consensus Vpr proteins were revealed by immunoblotting. The cells were harvested 24 h postaddition of doxycycline. U2OS cells not expressing Vpr (–) and U2OS-iH1vpr cells expressing the HIV-1 NL4-3 *vpr* allele (NL) provided negative and positive controls, respectively. Tubulin (Tub) provided loading controls.

To validate Exo1 as bona fide Vpr target, we began by testing whether Exo1 is depleted in HIV-1-infected CD4^+^ T cells. CD4^+^ T cells were isolated from human peripheral blood mononuclear cells by positive selection, activated with CD3/CD28 beads, and challenged 24 h later with single-cycle HIV-1 NL4-3-derived viruses possessing either an intact (NL4-3.GFP.*R*+) or disrupted (NL4-3.GFP.R–) *vpr* gene and expressing a green fluorescent protein (GFP) marker protein (16). Two days postinfection, the productively infected cells were isolated by cell sorting for GFP fluorescence, and Exo1 levels in lysates prepared from the sorted cells were assessed by immunoblotting. As shown in Fig. 1A, Exo1 levels were depleted in cells infected with HIV-1 harboring the intact, but not the disrupted, *vpr* gene. The infected cell lysates were also blotted for HLTF, MUS81, and UNG2, previously validated direct substrates of HIV-1 Vpr-CRL4^DCAF1^ E3 involved in postreplication DNA repair (16, 17, 31). The extent of Exo1 depletion in cells infected with HIV-1 expressing Vpr was comparable to that of HLTF and more pronounced than that seen for MUS81.

Next, we examined the kinetics of Exo1 depletion by Vpr and compared them to those of other Vpr-recruited substrates of Vpr-CRL4^DCAF1^ E3. To this end, U2OS-iH1vpr cells were induced with doxycycline to express Vpr and collected at various times postinduction. The levels of Vpr targets in cell lysates were subsequently characterized by immunoblotting. Figure 1B shows that Exo1 levels were depleted with kinetics similar to those seen for HLTF, in line with the data from primary CD4^+^ T cells. We conclude that HIV-1 infection depletes Exo1 levels in infected CD4^+^ T cells in a Vpr-dependent manner to an extent similar to that seen for previously validated targets of Vpr-CRL4^DCAF1^ E3.

### Exo1 is a conserved target of HIV-1 and SIVcpz lineage Vpr.

To assess the generality of our finding, we next tested Vpr proteins from the main groups of HIV-1 and closely related SIVcpzs, the latter persisting in chimpanzees (32). U2OS cell populations were engineered to inducibly express synthetic consensus Vpr proteins representative of HIV-1 groups M, N, and O as well as those representative of Vpr proteins encoded by two distinct populations of SIVcpzs isolated from two chimpanzee subspecies: Pan troglodytes *troglodytes* and Pan troglodytes *schweinfurthii*. The cells were induced to express Vpr, cell lysates were prepared 24 h postinduction, and Exo1 levels were characterized by immunoblotting (Fig. 1C). We found that all tested consensus HIV-1 and SIVcpz Vpr proteins depleted Exo1 levels, similar to what occurred with HIV-1 NL4-3 Vpr. We conclude that Exo1 is a common target of Vpr proteins of the HIV-1/SIVcpz lineage viruses.

### Exo1 depletion by Vpr is cell cycle phase independent.

**FIG 2:**
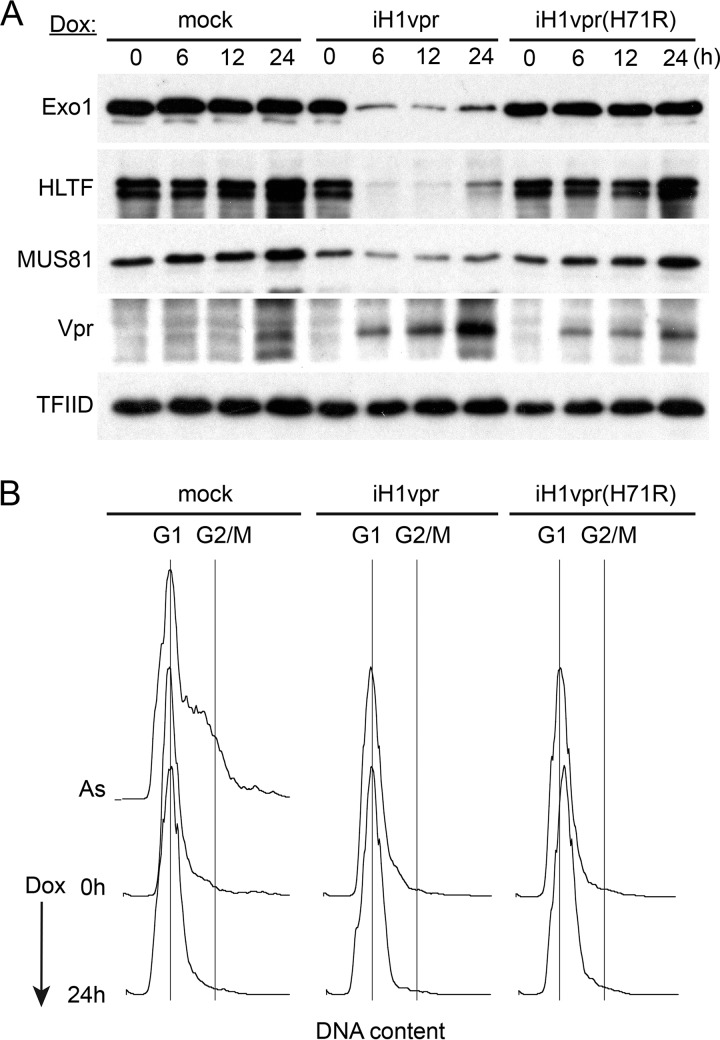
HIV-1 Vpr depletes Exo1 outside G_2_ phase. U2OS cells harboring doxycycline-inducible transgenes expressing wild-type HIV-1 NL4-3 Vpr (iH1vpr) or its H71R mutant [iH1vpr(H71R)], which does not bind CRL4^DCAF1^ E3, and control U2OS cells (mock) were synchronized by a double-thymidine block and kept arrested at the G_1_/S border for the duration of the experiment. Vpr expression was induced by addition of doxycycline (100 ng/ml) to the culture medium 8 h following the initiation of the second thymidine treatment, and cells were collected at the indicated time points for immunoblot analysis of Exo1 levels (A) and flow cytometric analysis of cell cycle position (B). Levels of HLTF and MUS81, two previously identified Vpr-recruited substrate proteins of CRL4^DCAF1^ E3, were also characterized, as a reference. TFIID was used as a loading control. DNA content profiles were characterized for the cell populations at the time of doxycycline addition (0 h) and at the last time point (24 h). A DNA profile obtained for an asynchronously dividing U2OS cell population (As) is shown as a reference. The abscissa is DNA content, revealed by 7AAD staining, shown on a linear scale. The positions of cells in G_1_ and G_2_/M phases are indicated.

Vpr induces cell cycle arrest at the G_2_-phase DNA damage checkpoint. To exclude the possibility that Exo1 levels are physiologically downregulated in G_2_ and the observed Exo1 depletion is an indirect consequence of the accumulation of Vpr-expressing cells at the G_2_ checkpoint, we asked whether Vpr can deplete Exo1 outside the G_2_ phase, as we showed previously for HLTF (16). To address this, U2OS-iH1vpr cells were synchronized at the G_1_/S border by double-thymidine block. Vpr expression was then induced with doxycycline 8 h following the initiation of the second thymidine treatment, and cells were harvested at different time points after doxycycline induction for determination of Exo1 levels by immunoblotting. As controls, we used parental U2OS cells and U2OS cells inducibly expressing Vpr with the H71R mutation [Vpr(H71R)], which disrupts Vpr binding to the DCAF1 substrate receptor subunit of the CRL4^DCAF1^ E3 complex (7). DNA profiles of cells collected at the time of initiation of the doxycycline treatment and at the 24-h time point clearly show that the cells remained arrested near G_1_/S transition throughout the duration of the experiment (Fig. 2). Exo1 levels were depleted within 6 h of the doxycycline induction of wild-type Vpr, similar to what occurred with those of HLTF. Significantly, the Vpr(H71R) mutant did not have such an effect. The finding that Vpr can deplete Exo1 at the G_1_/S border demonstrates that Exo1 depletion is not an indirect consequence of the Vpr-induced G_2_ arrest. The observation that Vpr(H71R) does not deplete Exo1 tentatively links the depletion to Vpr subversion of the CRL4^DCAF1^ E3 and ubiquitin proteasome system.

### Vpr depletes Exo1 by a proteasome-dependent mechanism that is independent of cyclin F (CCNF) and ATR.

**FIG 3:**
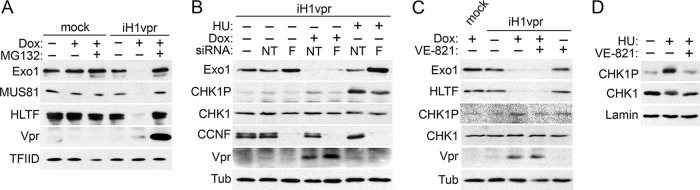
Vpr depletes Exo1 by a proteasome-dependent mechanism that is independent of CCNF and an ATR controlled checkpoint. (A) Vpr targets Exo1 for proteasome-dependent degradation. U2OS-iH1Vpr (iH1vpr) and control U2OS cells (mock) were treated, or not, with doxycycline (100 ng/ml) to induce Vpr expression in the absence or presence of the MG132 proteasome inhibitor (1 μg/ml) for 24 h, and cell extracts were analyzed by immunoblotting for the indicated proteins. FLAG-tagged Vpr was detected with an α-FLAG antibody. TFIID provided a loading control. (B) Exo1 depletion by Vpr is not mediated by CCNF. U2OS-iH1Vpr and control U2OS cells were subjected to RNAi targeting CCNF (lanes F) or nontargeting RNAi (lanes NT). Two days later, the cells were treated with doxycycline (100 ng/ml) and/or hydroxyurea (HU; 3 mM) for 24 h, and extracts were analyzed by immunoblotting for Exo1, Vpr, CCNF, total CHK1, and S345-phosphorylated CHK1 (CHK1P). Tubulin (Tub) provided a loading control. (C) Vpr-mediated Exo1 depletion is independent of the induction of the ATR-controlled checkpoint. U2OS-iH1Vpr and control U2OS cells were induced with doxycycline (100 ng/ml) in the presence or absence of the VE-821 ATR inhibitor (2 µM) for 24 h. Cell extracts were analyzed by immunoblotting for the indicated proteins. Tubulin provided a loading control. (D) VE-821 inhibits CHK1 phosphorylation. U2OS cells were cultured in the presence of hydroxyurea (3 mM) in the absence or presence of VE-821 (2 µM) for 24 h. Total and S345-phosphorylated CHK1 levels in cell extracts were revealed by immunoblotting. Lamin B1 (Lamin) provided a loading control.

Next, we asked whether Vpr targets Exo1 for degradation by a proteasome-dependent mechanism. To test this, U2OS-iH1vpr and control cells were cultured with doxycycline in the presence or absence of the MG132 proteasome inhibitor. Twenty-four hours postinduction, cell lysates were prepared and Exo1 levels, along with those for MUS81 and HLTF, were revealed by immunoblotting. As shown in Fig. 3A, proteasome inhibition prevented the depletion of Exo1 levels in Vpr-expressing cells.

Exo1 can be degraded by a proteasome-dependent mechanism in response to DNA damage and/or replication stress. In particular, following UV irradiation, Exo1 degradation is mediated by the SCF-CCNF E3 complex (33); further, following DNA double-stranded break induction, Exo1 is rapidly degraded by the ubiquitin-proteasome system via an ATR-controlled mechanism (34). Since Vpr expression induces hallmarks of the cellular response to damaged DNA (35, 36), we asked whether any of the above-described mechanisms mediates the Vpr-dependent depletion of Exo1.

To address the role of the SCF-CCNF, U2OS-iH1vpr cells were subjected to RNA interference (RNAi) targeting CCNF (F) or nontargeting (NT) RNAi and 2 days later induced to express Vpr. It is evident from the data shown in Fig. 3B that CCNF depletion did not restore Exo1 levels in cells expressing Vpr. Importantly, RNAi-mediated CCNF depletion did inhibit Exo1 degradation in response to hydroxyurea (HU)-induced replication stress. The latter control indicates that RNAi to CCNF was deep enough to disrupt SCF-CCNF E3-mediated Exo1 degradation.

We then asked whether ATR signaling is required for Vpr-induced Exo1 degradation. U2OS-iH1vpr cells were induced with doxycycline in the absence or presence of the VE-821 ATR kinase inhibitor. As shown in Fig. 3C, VE-821 treatment did not prevent Exo1 depletion in Vpr-expressing cells. Of note, the VE-821 treatment noticeably suppressed ATR-mediated CHK1 phosphorylation on serine S345 (CHK1P) (37). To corroborate the finding that VE-821 indeed inhibited ATR in this experiment, U2OS cells were exposed to HU, which activates the ATR-controlled checkpoint, leading to a much more robust CHK1 phosphorylation than that induced by Vpr, in the presence or absence of the same VE-821 concentration. Data in Fig. 3D clearly show that VE-821 prevented the HU-induced ATR-mediated CHK1 phosphorylation in this experimental setting. We conclude that the canonical degradation mechanisms linked to cellular response to damaged DNA are not likely to mediate HIV-1 Vpr-induced Exo1 degradation. These findings strengthen the possibility that Exo1 is a direct target of the hijacked by HIV-1 Vpr CRL4^DCAF1^ E3 ubiquitin ligase.

### Vpr recruits Exo1 to CRL4^DCAF1^ E3 for polyubiquitinaltion.

**FIG 4:**
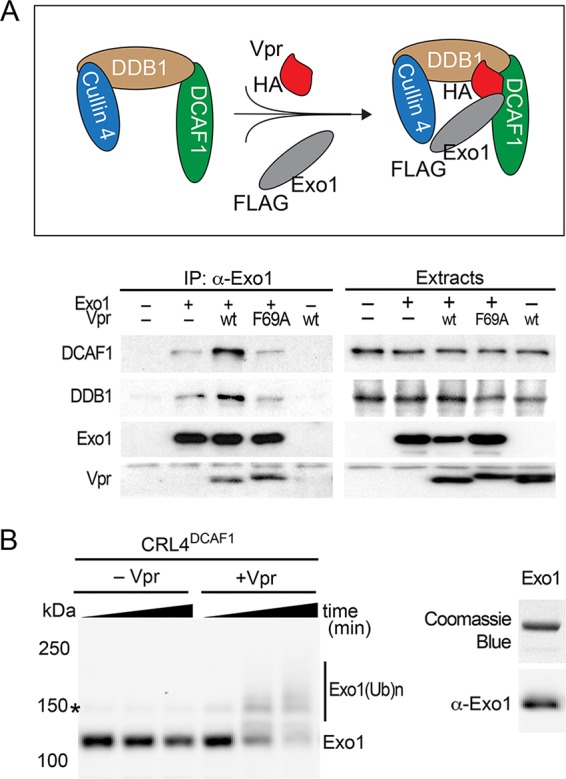
Vpr recruits Exo1 to the CRL4^DCAF1^ E3 for ubiquitination. (A) Vpr connects Exo1 to the endogenously expressed DDB1-DCAF1 module of the CRL4^DCAF1^ E3 complex *in vivo*. (Top) Schematic representation of Vpr-mediated recruitment of Exo1 to the CRL4^DCAF1^ E3 complex. The placement of HA and FLAG epitope tags on Vpr and Exo1, respectively, is indicated. (Bottom) FLAG-Exo1 was transiently coexpressed with HA-tagged wild-type HIV-1 Vpr (wt) or Vpr(F69A), which does not bind DCAF1, in HEK293T cells, as indicated. Endogenous DCAF1, DDB1, and ectopic Vpr and Exo1 were revealed in Exo1 immune complexes and in detergent extracts by immunoblotting. (B) Vpr-dependent Exo1 ubiquitination by recombinant CRL4^DCAF1^ E3 *in vitro*. (Left) *In vitro* ubiquitination assays were performed with recombinant Exo1 incubated with CRL4^DCAF1c^ E3 reconstituted from recombinant subunits, in the absence or presence of recombinant HIV-1 NL4-3 Vpr. Reactions were sampled over time, and native (Exo1) and ubiquitinated [Exo1(Ub)n] forms of Exo1 were revealed by immunoblotting with α-Exo1 antibody. An asterisk indicates a nonspecific band. (Right) Recombinant Exo1 expressed and purified from E. coli was analyzed by SDS-PAGE, gel stained with Coomassie blue (upper), and confirmed by immunoblotting with α-Exo1 antibody.

We next asked whether HIV-1 Vpr connects Exo1 to the endogenously expressed DCAF1 and DDB1 subunits of the CRL4^DCAF1^ E3 complex (Fig. 4A and B). FLAG-tagged Exo1 was expressed in HEK293T cells, alone or with hemagglutinin epitope (HA)-tagged wild-type HIV-1 NL4-3 Vpr or the Vpr(F69A) variant, which does not bind DCAF1 (12). Anti-FLAG-Exo1 immune complexes were isolated from lysates prepared from the transfected cells and immunoblotted for Exo1, DDB1, DCAF1, and Vpr. We found that wild-type Vpr promoted the assembly of a protein complex containing Exo1 and the DCAF1-DDB1 module of the E3 complex. In contrast, no such effect was observed with the Vpr(F69A) mutant, which does not bind DCAF1, even though this mutant associated with Exo1 to a degree similar to that of wild-type Vpr. Low levels of DCAF1 and DDB1 that coprecipitated with Exo1 in the presence of Vpr(F69A) were similar to those seen when Exo1 complexes were formed in the absence of Vpr. Whether the latter reflects a residual nonspecific association of the immune complexes with Exo1 or a low level of Exo1 binding to DDB1-DCAF1 in the absence of Vpr is not yet known. Regardless, our data clearly show that wild-type Vpr specifically recruits Exo1 to the CRL4^DCAF1^ E3 complex, via its binding to the DCAF1 subunit.

To corroborate and extend the above findings, we asked whether Exo1 is a Vpr-dependent substrate of the CRL4^DCAF1^ E3 ubiquitin ligase, using an *in vitro* ubiquitination system reconstituted with recombinant proteins (13, 31). As shown in Fig. 4B, CRL4^DCAF1^ E3 ligase in complex with Vpr mediated polyubiquitination of Exo1. In contrast, no polyubiquitinated species of Exo1 were observed upon incubation of Exo1 with E3 ligase alone. These data provide further support to a model in which Vpr directly loads Exo1 onto the CRL4^DCAF1^ E3 ligase for polyubiquitination and subsequent degradation by the proteasome.

### Exo1 depletion alone does not explain Vpr’s ability to arrest cells in the G_2_ cell cycle phase.

**FIG 5:**
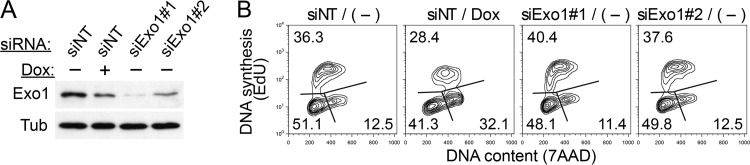
Exo1 depletion alone does not account for the ability of Vpr to arrest cells in the G_2_ cell cycle phase. U2OS-iH1vpr cells were subjected to RNAi with siRNAs targeting two distinct regions in Exo1 mRNA (siExo1#1 and #2) or a nontargeting RNAi (siNT). Twenty-four hours postinitiation of RNAi, doxycycline (100 ng/ml) was added, or not, to the culture medium, and 24 h later, the cells were harvested for immunoblot analysis of Exo1 levels (A) or cultured in the presence of ethynyl deoxyuridine (EdU) for 30 min to label S-phase cells actively replicating their DNA and then harvested for flow cytometry analysis of cell cycle profiles (B). (B) The percentage of cells in G_1_, S, and G_2_/M phase are indicated. The abscissa is DNA content, revealed by 7AAD staining, shown on a linear scale. The ordinate is DNA synthesis, revealed by incorporation of EdU, shown on a logarithmic scale.

HIV-1 Vpr activates the DNA damage checkpoint that is controlled by the ATR kinase, leading to cell cycle arrest in the G_2_ phase (27). This effect might reflect Vpr antagonism of a protein(s) mediating postreplication DNA repair (16). Since the exact mechanism leading to checkpoint activation by Vpr is not yet known, we asked whether Exo1 depletion by Vpr plays a role. To this end, U2OS-iH1vpr cells were induced to express Vpr or subjected to RNAi with small interfering RNAs (siRNAs) targeting two distinct regions in Exo1 mRNA. The cells were harvested 48 h later, and Exo1 levels in cell lysates were assessed by immunoblotting and cell cycle profiles characterized by flow cytometry. As shown in Fig. 5, Exo1 levels were reduced following induction of Vpr expression, and the cells exhibited an altered cell cycle distribution with decreased G_1_ and S and increased G_2_/M cell populations, characteristics of the HIV-1 Vpr-induced cell cycle perturbations. Remarkably, even though Exo1 levels following RNAi were considerably lower than those seen in Vpr-expressing cells, RNAi-mediated Exo1 depletion was not sufficient to copy the Vpr-induced cell cycle arrest phenotype. Thus, Vpr-mediated Exo1 depletion alone appears insufficient to mediate deregulation of the cell cycle resulting from Vpr expression.

### Vpr targets Exo1’s C terminus via the Exo1 PCNA-interacting protein (PIP) binding motif.

**FIG 6:**
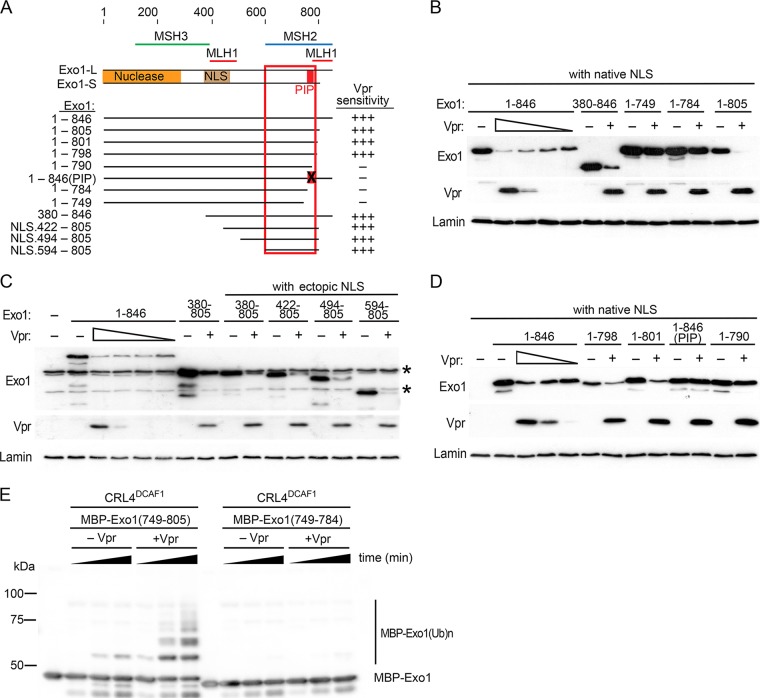
Vpr interacts with the Exo1 C-proximal region that binds MSH2. (A) Schematic representation of the Exo1 protein, the tested Exo1 mutants, and their effects on Vpr-mediated depletion of Exo1 levels. Functional organization of the two Exo1 isoforms (Exo1-L and -S) and Exo1 deletion mutants, including location of the nuclease domain, native nuclear localization signal (NLS), and regions mediating binding with the MSH2, MSH3, and MLH1 mismatch repair proteins are shown. A PIP box, located within the MSH2 binding domain, is represented by a red rectangle. The positions of the first and last amino acid residues are indicated for each deletion mutant. The residue 1 to 846 (PIP) variant is a full-length Exo1L with 788**Q**IK**L**NE**LW**→788**A**IK**A**NE**AA** PIP box mutations. The region of Exo1 that contributes to Vpr sensitivity is boxed. Exo1 deletion variants lacking the native NLS were tagged with the SV40 NLS (NLS) at their N terminus, as indicated. (B to D) Vpr interacts with an Exo1 region that comprises the PIP box and mediates binding to MSH2. FLAG-tagged Exo1 and its variants were transiently expressed alone (–) or coexpressed with FLAG-tagged HIV-1 NL4-3 Vpr (+) in HEK293T cells. Cell lysates were prepared 24 h posttransfection, and Exo1 and Vpr levels were revealed by immunoblotting with α-FLAG antibody. An asterisk indicates a nonspecific background band. (E) Vpr targets the PIP box of Exo1 to directly load it onto the CRL4^DCAF1^ E3. A recombinant MBP-Exo1 fusion protein containing Exo1 residues 749 to 805 [MBP-Exo1(749–805)] or residues 749 to 784 [MBP-Exo1(749–784)] were subjected to *in vitro* ubiquitination assays with the CRL4^DCAF1^ or Vpr-CRL4^DCAF1^ E3 ligase. The reaction mixtures were separated by SDS-PAGE and analyzed by immunoblotting with anti-MBP antibody.

To determine whether Vpr targets a known functional element within the Exo1 molecule, we next mapped the Exo1 region that mediates the effect of Vpr. A set of N- and C-terminal Exo1 deletion mutants were generated by (i) removing the N-terminal nuclease domain, (ii) removing a C-terminal region that is unique to the longer (L) of the two Exo1 isoforms, or (iii) deleting/disrupting domains that mediate binding with the mismatch repair proteins MLH1, MSH2, and MSH3, as well as additional regions whose functional roles have not yet been established (Fig. 6A) (30). Since several of the deletions removed the native Exo1 nuclear localization signal (NLS) and Exo1 interaction with Vpr and CRL4^DCAF1^ E3 probably occurs in the nuclear compartment, we restored the nuclear targeting of the variant proteins by fusing them to the well-characterized simian virus 40 (SV40) large T antigen NLS.

As shown in Fig. 6B, the Exo1(1–805) variant, corresponding to the shorter Exo1 isoform, was fully sensitive to Vpr expression, whereas the Exo1(1–784) mutant was resistant. This indicates that Vpr targets both Exo1 isoforms for degradation and that Exo1 sequence between residues 784 and 805 was required for the effect of Vpr. Analyses of additional mutants, shown in Fig. 6C, revealed that the N-proximal Exo1 region spanning the nuclease domain, MSH3, and the MLH1 binding site N-proximal segment was dispensable. This narrowed down the Exo1 region required for susceptibility to Vpr to residues located between positions 594 and 805 (Fig. 6C, left). This region comprises part of the Exo1 MSH2 binding element and a putative PCNA-binding motif (PIP box, residues 788 to 795) (38). Interestingly, Exo1 variants with C-terminal deletions that disrupt the integrity of the PIP box (1 to 790) (Fig. 6D), or completely delete it (1 to 784, 1 to 749) (Fig. 6B), were not depleted by Vpr. To directly address the possibility that Vpr targets Exo1 via its PIP box, we tested the effect of alanine substitution for hydrophobic residues in the PIP box (788**Q**IK**L**NE**LW**→788**A**IK**A**NE**AA**) in the context of full-length Exo1 protein [Exo1.1–846(PIP)]. Significantly, the levels of Exo1.1-846(PIP) mutant were not downregulated by Vpr (Fig. 6C, right). We conclude that the PIP box is necessary for Exo1 binding by Vpr and may constitute the Vpr binding site.

To further validate that the PIP box is directly targeted by Vpr, we subjected two MBP- Exo1 fusion peptides (residues 749 to 805 and 749 to 784) to *in vitro* ubiquitination assays with CRL4^DCAF1^ E3 reconstituted with or without Vpr (Fig. 6E). The fusion peptide containing the PIP box [MBP-Exo1(749–805)] was robustly polyubiquitinated by the CRL4^DCAF1^ E3 only when in complex with Vpr. In contrast, the peptide without the PIP box [MBP-Exo1(749–784)] was not ubiquitinated. These data strongly support the idea that Vpr interacts with Exo1 via its PIP box to recruit Exo1 onto the CRL4^DCAF1^ E3 ligase for subsequent proteasome-dependent degradation.

### Exo1 restricts HIV-1 infection.

To test whether Exo1 inhibits HIV-1 replication, we depleted endogenous Exo1 levels by RNAi in CEM.SS T cells harboring a doxycycline-inducible RNAi-resistant Exo1 gene (CEM.SS-iExo1r). Specifically, the cells were transduced with a retroviral vector expressing either a control nontargeting (NT) short hairpin RNA (shRNA) or an Exo1-targeting, miR30E-based shRNA and left untreated (Dox–) or treated (Dox+) with doxycycline to induce expression of RNAi-resistant Exo1 cDNA. Three days later, the cells were challenged with replication-competent HIV-1 NL4-3 virus possessing wild-type HIV-1 *vpr* [HIV(vpr.wt)] or inactivated *vpr* [HIV-1(vpr.Q8*)] at equivalent low multiplicities of infection (MOI). Viral replication was subsequently measured 7 days after HIV infection by real-time PCR quantification of HIV-1 DNA in the infected cultures and normalized to total cellular DNA.

**FIG 7:**
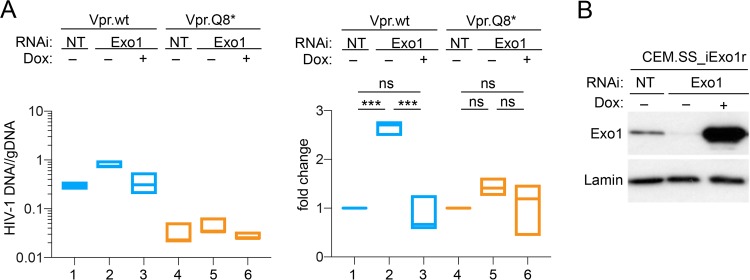
Exo1 restricts HIV-1 infection in T cells. (A) Exo1 restricts HIV-1 replication in CEM.SS T cells. CEM.SS_iExo1r T cells were subjected to RNAi nontargeting (NT) (boxes in lanes 1 and 4) or targeting Exo1 (boxes in lanes 2 and 5) in the absence (boxes 1, 2, 4, and 5) or presence (boxes 3 and 6) of doxycycline (100 ng/ml). Three day later, the cells were infected with HIV-1(vpr.wt) (blue box) or HIV-1(vpr.Q8*) (orange box) at MOI of 0.005 to 0.02. The cells were subcultured on days 3 and 5 and harvested 7 days after HIV-1 infection. Fewer than 5% of the cells expressed the RFP marker at the time of harvest. The levels of HIV-1 DNA, quantified by real-time PCR, are shown expressed as numbers of copies per cell genome equivalent (left). The fold changes in HIV-1 copy number in Exo1 depleted by RNAi (Exo1, lanes –) and expressing ectopic RNAi-resistant Exo1r (Exo1, lanes +) normalized to those in CEM.SS T cells subjected to nontargeting RNAi (NT) are also shown (right). Data from three biological replicates are shown as floating bars with line at median. ***, *P* < 0.001; ns (not significant), *P* > 0.05 compared with NT RNA (one-way ANOVA with Tukey’s *post hoc* test). (B) Exo1 levels in CEM.SS T cell populations at the time of HIV-1 infection were analyzed by immunobloting. Exo1 knockdown was stable over the duration of the experiment (not shown). Lamin B1 provided a loading control.

As shown in Fig. 7A, the intact wild-type *vpr* gene conferred an approximately 10-fold replication advantage in CEM.SS_iExo1r cells subjected to the control nontargeting RNAi over a 7-day period, compared to the vpr.Q8* condition (compare box 1 with box 4). We estimate that Vpr stimulated HIV-1 replication in CEM.SS T cells by approximately 20% to 40% per infection cycle. Of note, detection of such a modest, yet reproducible, effect was made possible by selecting an early assay endpoint, well before meaningful depletion of target cells and because HIV-1 DNA levels were quantified relative to cellular DNA.

The HIV-1(vpr.wt) virus replicated to higher levels in Exo1-depleted CEM.SS_iExo1r cells than in control NT cells over a 7-day period (∼3-fold, *P* < 0.00005) (Fig. 7A, right, boxes 1 and 2). This effect was also noted for HIV-1(vpr.Q8*) though less pronounced and not statistically significant using one-way analysis of variance (ANOVA) (∼1.5-fold, *P* > 0.05) (Fig. 7A, right, boxes 4 and 5). Of note, the Exo1-depleted and control CEM.SS_iExo1r cells proliferated at similar rates; this excluded the possibility that differences in HIV-1 DNA levels reflected a difference in the proliferation rates of the cells subjected to nontargeting versus Exo1-targeting RNAi (Fig. S2). Importantly, ectopic expression of the RNAi-resistant Exo1 variant restored restriction of HIV-1 infection in the endogenous Exo1-depleted CEM.SS_iExo1r cells (Fig. 7A, left and right, Dox+, boxes 3 and 6). This ruled out a potential involvement of an off-target effect of RNAi on Exo1 upon HIV-1 replication. We conclude that Exo1 restricts HIV-1 replication and that this restriction is partially counteracted by HIV-1 Vpr.

10.1128/mBio.01732-18.2 FIG S2 Exo1 depletion in CEM.SS T cells does not affect cell cycle distribution and proliferation rate. (A) CEM.SS T cells subjected to nontargeting (NT) or Exo1-targeting (Exo1) RNAi were stained with CellTracker Green CMFDA, and cell fluorescence, characterized by flow cytometry at the indicated time points postlabeling, was assessed. (B) Cell cycle profiles were determined 3 days postinitiation of RNAi. Download FIG S2, TIF file, 0.6 MB. Copyright © 2018 Yan et al. 2018 Yan et al. This content is distributed under the terms of the Creative Commons Attribution 4.0 International license.

Significantly, it is evident from the data shown in Fig. 7A (left panel, box 5 versus box 1) that Exo1 depletion did not restore replication of HIV-1(vpr.Q8*) to levels seen for HIV-1(vpr.wt). Furthermore, Exo1 depletion relieved the replication of HIV-1(vpr.Q8*) to a much smaller extent than that seen for HIV-1(vpr.wt), the former remaining largely restricted (Fig. 7A, right, ∼1.5-fold versus ∼3.0-fold, respectively). These data are best explained by a model in which HIV-1 replication in CEM.SS T cells is restricted not only by Exo1 but also by one or more additional mechanisms which are also counteracted by Vpr. In such a case, in Exo1-depleted cells, vpr-deficient HIV-1(vpr.Q8*) remains restricted by these additional mechanisms, whereas HIV-1(vpr.wt) is relieved to a larger extent due to the presence of functional *vpr*, which counteracts them.

## DISCUSSION

Viral accessory virulence factors hijack host cell E3 ubiquitin ligases to disable innate/intrinsic defenses and thereby provide a more permissive environment for virus replication. Here, we show that Vpr proteins of HIV-1 and its ancestor SIVcpz lineage reprogram CRL4^DCAF1^ E3 to antagonize the postreplication DNA repair enzyme Exo1. We show that Vpr recruits Exo1 to the CRL4^DCAF1^ E3 complex for ubiquitination and subsequent proteasome-dependent degradation, and we corroborate this model by excluding the possibility that previously identified CRL4^DCAF1^ E3-independent mechanisms are involved in Vpr-mediated Exo1 degradation. Finally, our data show that Exo1 exerts a modest inhibitory effect on HIV-1 replication in T cells, consistent with the model in which Vpr reprograms CRL4^DCAF1^ E3 to counteract this restriction. Notably, Exo1 is the fourth postreplication DNA repair enzyme, along with the previously described UNG2, MUS81, and HLTF, that is antagonized by HIV-1 Vpr via CRL4^DCAF1^ E3 (13, 31, 39). Together, these findings reveal that HIV-1 Vpr extensively remodels the cellular postreplication DNA repair machinery by impinging on multiple repair pathways and thereby promotes HIV-1 replication.

Our studies identify Exo1 as a direct target of HIV-1 Vpr for recruitment to CRL4^DCAF1^ E3 and subsequent proteasome-dependent degradation. Several lines of evidence support direct binding of Vpr with Exo1. First, we find that levels of known Exo1 interaction partners, including the mismatch repair proteins MSH2, MSH3, and MLH1 (30) and subunits of the MRN complex (40), which recruits Exo1 for DNA end resection, are unaltered in Vpr-expressing cells (see Fig. S1 in the supplemental material), consistent with the possibility that Vpr targets Exo1 alone, rather than in a complex with any of the above proteins. Second, Exo1 regions that comprise binding sites for its known interaction partners, with the exception of PCNA, are also dispensable for the effect of Vpr. Regarding PCNA, our data clearly show that Exo1 PIP box mutants resist depletion by Vpr in cell-based assays (Fig. 6). Although this finding allows for the possibility that PCNA bridges Vpr binding with Exo1, we also show that Vpr can load full-length Exo1 as well as the PIP box onto the CRL4^DCAF1^ E3 complex for polyubiquitination in the absence of PCNA in a defined *in vitro* system. Furthermore, we observed previously that PCNA levels are unaltered in HIV-1 Vpr-expressing cells under conditions of Exo1 depletion (16); this again favors the possibility that Vpr targets Exo1 independently of PCNA. Overall, our evidence supports the model in which Vpr binds Exo1 directly, via its PIP box, for loading onto the CRL4^DCAF1^ E3 complex.

The Exo1 PIP box mediates Exo1 loading at sites of DNA damage by PCNA (38). In this context, it is interesting to note that HIV-1 Vpr captures UNG2 and HLTF via their DNA binding domains by mimicking DNA (13, 39). Thus, it appears that Vpr loads its target DNA repair proteins onto the CRL4^DCAF1^ E3 complex by binding to their structured elements that mediate recruitment to sites of DNA damage. Notably, the strategy of antagonizing innate immune proteins by mimicking binding of their natural functionally important ligands minimizes the chances of selecting escape mutations, which would likely compromise the binding surface functionality.

The results of our spreading infection assays in Exo1-depleted CEM.SS T cells clearly show that Exo1 inhibits HIV-1 replication. It is noteworthy that, upon Exo1 depletion, the increase in replication of HIV-1(vpr.Q8*), lacking functional *vpr*, was relatively small compared to that seen for HIV-1(vpr.wt), indicating that *vpr*-deficient HIV-1 remained largely restricted. This finding would not be expected if Exo1-mediated restriction were the only Vpr-counteracted mechanism inhibiting HIV-1 replication in these cells. Although we cannot exclude the possibility that this was due to incomplete Exo1 depletion by RNAi, overall, our evidence points to a role for additional restrictions on HIV-1 replication that are counteracted by Vpr.

One could speculate, based on the available data, that Exo1 restricts an early step(s) in HIV-1 infection. Vpr is incorporated into HIV-1 virions in producer cells and, thus, is present in target cells during early postentry events (41). Notably, HIV-1 plus-strand reverse transcripts are primed from multiple primers (42, 43) and contain 5′ flaps, gaps, and RNA/DNA hybrids, which resemble canonical substrates processed by Exo1 5′–3′ exonuclease, flap nuclease, and/or RNase H (30). There are no known checkpoints that would prevent incompletely reverse transcribed HIV-1 genomes from being imported into the interphase nucleus, where they can be accessed by cellular postreplication DNA repair machinery. Hence, we speculate that virion-carried Vpr antagonizes Exo1 to prevent processing of HIV-1 reverse transcripts and/or integration intermediates, as such processing (specifically by Exo1) would interfere with the ordered progression of HIV-1 reverse transcription and/or repair at provirus integration sites and thereby directly inhibit early steps in HIV-1 infection. It is also possible that Vpr depletes Exo1 in producer cells to prevent Exo1 incorporation into the HIV-1 virion for restricting the infection in the target cell. We do not favor this possibility, however, because Exo1 levels are not detectable by immunoblotting in HIV-1 virions produced in the absence of Vpr (M. C. Shun and J. Skowronski, unpublished data).

The failure of RNAi-mediated Exo1 depletion to restore replication of *vpr*-deficient HIV-1(vpr.Q8*) to levels seen for HIV-1(vpr.wt) is evidence that Exo1 is not the only Vpr-antagonized protein inhibiting HIV-1 infection in CEM.SS T cells. Obvious candidates are suggested by previous findings that HIV-1 Vpr antagonizes HLTF and MUS81-EME1(EME2) postreplication DNA repair enzymes and TET2, also via CRL4^DCAF1^ E3. It is worth noting that the former antagonisms are conserved functions of Vpr proteins of HIV-1/SIVcpz lineages (16), like the antagonism of Exo1. Unlike Exo1, HLTF and MUS81 process generic branched DNA structures, such as those transiently generated during HIV-1 reverse transcription (42, 43). Hence, they could partner with Exo1, each processing a distinct set of HIV-1 replication intermediates, to restrict infection. Regardless of the exact mechanism, the coordinated removal of a subset of enzymes that process branched DNA structures via Vpr-CRL4^DCAF1^ E3 supports the notion that they are also likely to impinge on HIV-1 replication.

It is conceivable that the restrictions exerted by the DNA repair enzymes antagonized by Vpr may be more robust in specific cell types and/or when reverse transcription is suboptimal, due to exceedingly low canonical dNTP concentrations, such as in monocyte-derived cell lineages and/or quiescent/minimally activated CD4^+^ T cells, or when other constraints are placed by intrinsic-recognition HIV-1 reverse transcripts. Such conditions, once identified, might provide a more robust experimental system(s) for follow-up virologic/mechanistic studies. Since the Vpr antagonism of postreplication DNA repair enzymes is conserved across HIV-1/SIVcpz clades, there is little doubt that cellular DNA repair machinery plays an important, yet poorly understood, role in HIV-1 replication.

## MATERIALS AND METHODS

### Ethics statement.

This study was approved by the University Hospitals Institutional Review Board (IRB) (IRB number 12-09-23).

### Expression constructs and genes.

HIV-1 group M, N, and O, *P. troglodytes troglodytes* SIVcpz, and P. troglodytes *schweinfurthii* SIVcpz consensus *vpr* genes were previously described (16). FLAG-AU1-tagged *vpr* cDNAs were subcloned into the pLVX TetOne Puro vector (Clonetech). The human Exo1 cDNA was amplified by PCR from an EST clone (MHS6278-202826750; Open Biosystems) and subcloned into the pCG vector with an N-terminal myc, HA, or FLAG-AU1 epitope tag. Exo1 deletion mutants were constructed by PCR. Those lacking the native NLS sequence were tagged at their N terminus with a peptide comprising the FLAG-AU1 epitope tag followed by the SV40 NLS sequence and a GA di-amino acid linker (MADYKDDDDKGDTYRYIGAGARPKKKRKVGAR). An RNAi-resistant Exo1r gene was constructed by introducing silent mutations into Exo1 sequences targeted by Exo1mir30E.33 (ACGACAAGCCAATCTTCTT→ACGCCAGGCTAACCTCCTC) and Exo1mir30E.34 (AGATGTAGCACGTAATTCA→AGACGTTGCTCGAAACTCT) and cloned between BamHI and XhoI sites in the pEasiLv-puro polylinker.

### HIV-1 reporter viruses and retroviral vectors.

Single-cycle HIV-1 NL4-3.GFP.*R*+ and NL4-3.GFP.R– viruses were previously described (16). HIV-1(vpr.Q8*) was generated by mutating Vpr Q8 to a stop codon (CAA→TAA) in HIV-1 NL4-3.GFP.*R*+. Replication-competent HIV-1(vpr.wt) and HIV-1(vpr.Q8*) were constructed by substituting the tag red fluorescent protein (RFP) gene for the GFP gene and restoring the intact NL4-3 *env* gene. Virus particles were produced from HEK293T cells, concentrated, and normalized by infectivity to Jurkat T cells and by quantitative Gag immunoblotting, as described previously (44, 45). pLVX-TRE3G and pLVX-Tet-One Puro-inducible expression vectors (Clontech) were produced from HEK 293T cells as described previously (16). pEasiLv-puro was constructed by replacing the SpeI-SalI fragment comprising the E2-Crimson gene in pEasiLv (46) with the puromycin *N*-acetyl-transferase gene. All mutations and constructs were verified by DNA sequencing.

### Cell lines.

HEK293T, U2OS, and CEM.SS T cells (47, 48), the last obtained through NIH AIDS Reagent Program, were maintained in DMEM or RPMI1640 as previously described (16). U2OS cells expressing doxycycline-inducible HIV-1 and SIVcpz Vpr proteins were constructed using the pLVX Tet-One Puro vector (Clontech) and maintained under puromycin selection (2 μg/ml). Vpr expression was induced by doxycycline (100 ng/ml; Sigma-Aldrich) addition to the culture media. CEM.SS_iExo1r cells harbor the doxycycline-inducible RNAi-resistant Exo1r gene.

### RNAi.

RNAi in U2OS cells was performed as previously described (16, 44) using the following siRNAs: Exo1#1 (5′-UGCCUUUGCUAAUCCAAUCCCACGC-3′ [49]; Invitrogen catalog number HSS113557), Exo1#2 (5′-CCACCUAGGACGAGAAAUA-3′ [50]; Dharmacon catalog number J-013120-07), cyclin F (5′-CCAGUUGUGUGCUGCAUUA-3′ [33]) and (5′-UAGCCUACCUCUACAAUGA-3′ [51]). The latter two siRNA duplexes and the control nontargeting (NT) siRNA pool (D-001810-10) were also purchased from Dharmacon. Stable RNAi was performed with retroviral MSCV.eGFP.mir30E vectors expressing NT or Exo1-specific Exo1.33 and Exo1.34-enhanced mir30E shRNAs (52). mir30E shRNAs were constructed to the following sequences: NT, TAAGGCTATGAAGAGATAC; Exo1.33, GACGACAAGCCAATCTTCTTA; and Exo1.34, CAGATGTAGCACGTAATTCAA. The cells were transduced with retroviruses expressing individual nontargeting mir30E or a mixture of Exo1 targeting mir30E shRNAs at a combined MOI of ∼3.0 and challenged with HIV-1 NL4-3 3 days postinitiation of RNAi.

### Isolation of HIV-1-infected primary CD4^+^ T cells.

CD4^+^ T cells were purified from human PBMCs by negative selection (16). The cells, 1 × 10^6^ cells/well in a 96-well plate, were activated with Dynabeads human T-Activator CD3/CD28 (Invitrogen) in the presence of IL-2 (30 U/ml) in RPMI 1640 medium supplemented with 10% FBS, penicillin, and streptomycin. Two days later, cells were infected with a single-cycle HIV-1 NL4-3.GFP.*R*+ or NL4-3.GFP.R– virus. Forty-eight hours postinfection, productively infected GFP-positive cells were collected and suspended in PBS supplemented with BSA (1%), and live productively infected GFP-positive cells were isolated by sorting on a FACS Aria. Whole-cell lysates prepared from sorted cells were analyzed by immunoblotting.

### Cell synchronization and cycle analysis.

U2OS-iH1vpr cells were synchronized in early S phase by double-thymidine block and cell cycle analyses performed as described previously (16).

### Purification of Exo1 immune complexes.

HEK293T cells were transiently cotransfected with plasmids expressing HA-Vpr and FLAG-Exo1 by the calcium phosphate coprecipitation method (53). Protein complexes were purified from whole-cell extracts by precipitations via a FLAG epitope tag followed by competitive elution with FLAG epitope peptide under native conditions, as we previously described (7, 53).

### Protein expression, purification, and *in vitro* ubiquitination assays.

The Exo1 construct cloned into pET28 vector (EMD Biosciences) with His_6_ at the C terminus was expressed in Escherichia coli Rosetta 2 (DE3) in autoinduction medium at 18˚C for 16 h. Proteins were purified using a 5 ml Ni-NTA column (GE Healthcare), and Hi-Load Superdex200 gel-filtration column (GE Healthcare) equilibrated with a buffer containing 25 mM sodium phosphate, pH 7.5, 150 mM NaCl, 1 mM DTT, 5% glycerol, and 0.02% sodium azide. Purified proteins were confirmed by anti-Exo1 antibody. GST coding sequence in pET41 vector (EMD Biosciences) was replaced with MBP cDNA and two Exo1 peptides (residues 749 to 805 and 749 to 784) with C-terminal His_6_ tag were fused to MBP. MBP-fusion proteins were purified as described above. All other proteins used in *in vitro* ubiquitination assays were prepared as described previously (31, 54). The assays were performed as described previously (13). The extent of Exo1 ubiquitination was assessed by immunoblotting with α-Exo1 antibody or α-MBP antibody.

### Immunoblotting and antibodies.

Whole-cell extracts were separated by SDS-PAGE and transferred to a PVDF membrane for immunoblotting, performed as previously described (7). Protein bands were revealed by enhanced chemiluminescence or with fluorescent secondary antibodies (KPL) and an Odyssey infrared imager (Licor). The following antibodies were used: anti-UNG2 (α-UNG2; TA503563) from OriGene; α-SMUG1 (R1615-1), α-TDG (A304-365A-T), α-CCNF (A303-406A), α-XRCC1 (A300-065A-T), α-RNASEH2A (A304-149A-T), α-Top1 (A302-590A-T), α-MSH2 (A300-452A-T), α-MSH3 (A305-314A-T), α-XPA (A301-780A-T), α-XPC (A301-122A-T), α-XPD (A303-658A-T), α-RAD23A (A302-880A-T), α-RAD23B (A302-306A-T), α-XPB/ERCC3 (A301-337A-T), α-XPF/ERCC4 (A301-315A-T), α-ERCC5/XPG (A301-484A-T), α-NBS1 (A301-2891-T), α-Rad50 (A300-185A-T), α-Mre11 (A303-998A-T), α-CtIP (A300-488A-T), α-exonuclease 1 (A302-640A-T or A302-639A), α-BLM (A300-110A-T), α-Fen1 (A300-255A-T), and α-HLTF (A300-230A) from Bethyl Laboratories Inc.; α-Ape1 (4128), α-MSH6 (5424), α-MLH1 (3515), α-ERCC1 (12345), and α-CHK1 Ser345P (2341P) from Cell Signaling; α-RNAseH2b (sc-84590), α-RNAseH2c (sc-68228), α-Mus81 (sc-53382), α-CHK1 (sc-8408), α-α-tubulin (sc-5286), α-DCAF1 (sc-376850), and α-TFIID (sc-204) from Santa Cruz Biotechnology; α-DNA2 (ab194939) and α-Lamin B1 (ab16048) from Abcam; α-FLAG (F1804) from Sigma-Aldrich; and α-MBP (50-811-585) from New England Biolab. α-HA (12CA5 [55]), α-DDB1 (number 37-6200) from Invitrogen, and α-SF2 was provided by A. Krainer. α-Myc (9E10) and α-HIV-1 CA (183-H12-5C) were produced in house as we described previously (16). The 183-H12-5C hybridoma was obtained through the NIH AIDS Reagent Program, Division of AIDS, National Institute of Allergy and Infectious Diseases, NIH, from Bruce Chesebro (56).

### Statistical analyses.

The statistical significance of the data was analyzed using one-way ANOVA with the *post hoc* Tukey test, and graphs were prepared using PRISM 7.0 (GraphPad Software, La Jolla, CA).

## References

[1] Sauter D, Kirchhoff F 2018 Multilayered and versatile inhibition of cellular antiviral factors by HIV and SIV accessory proteins. Cytokine Growth Factor Rev 40:3–12. doi:10.1016/j.cytogfr.2018.02.005. 29526437

[2] Ulane CM, Kentsis A, Cruz CD, Parisien JP, Schneider KL, Horvath CM 2005 Composition and assembly of STAT-targeting ubiquitin ligase complexes: paramyxovirus V protein carboxyl terminus is an oligomerization domain. J Virol 79:10180–10189. doi:10.1128/JVI.79.16.10180-10189.2005. 16051811 PMC1182666

[3] Viswanathan K, Fruh K, DeFilippis V 2010 Viral hijacking of the host ubiquitin system to evade interferon responses. Curr Opin Microbiol 13:517–523. doi:10.1016/j.mib.2010.05.012. 20699190 PMC2939720

[4] Desimmie BA, Delviks-Frankenberrry KA, Burdick RC, Qi D, Izumi T, Pathak VK 2014 Multiple APOBEC3 restriction factors for HIV-1 and one Vif to rule them all. J Mol Biol 426:1220–1245. doi:10.1016/j.jmb.2013.10.033. 24189052 PMC3943811

[5] Margottin F, Bour SP, Durand H, Selig L, Benichou S, Richard V, Thomas D, Strebel K, Benarous R 1998 A novel human WD protein, h-beta TrCp, that interacts with HIV-1 Vpu connects CD4 to the ER degradation pathway through an F-box motif. Mol Cell 1:565–574. doi:10.1016/S1097-2765(00)80056-8. 9660940

[6] Jain P, Boso G, Langer S, Soonthornvacharin S, De Jesus PD, Nguyen Q, Olivieri KC, Portillo AJ, Yoh SM, Pache L, Chanda SK 2018 Large-scale arrayed analysis of protein degradation reveals cellular targets for HIV-1 Vpu. Cell Rep 22:2493–2503. doi:10.1016/j.celrep.2018.01.091. 29490283 PMC5916846

[7] Hrecka K, Gierszewska M, Srivastava S, Kozaczkiewicz L, Swanson SK, Florens L, Washburn MP, Skowronski J 2007 Lentiviral Vpr usurps Cul4-DDB1[VprBP] E3 ubiquitin ligase to modulate cell cycle. Proc Natl Acad Sci U S A 104:11778–11783. doi:10.1073/pnas.0702102104. 17609381 PMC1906728

[8] Angers S, Li T, Yi X, MacCoss MJ, Moon RT, Zheng N 2006 Molecular architecture and assembly of the DDB1-CUL4A ubiquitin ligase machinery. Nature 443:590–593. doi:10.1038/nature05175. 16964240

[9] Romani B, Cohen EA 2012 Lentivirus Vpr and Vpx accessory proteins usurp the cullin4-DDB1 (DCAF1) E3 ubiquitin ligase. Curr Opin Virol 2:755–763. doi:10.1016/j.coviro.2012.09.010. 23062609 PMC3955192

[10] Zhang S, Feng Y, Narayan O, Zhao LJ 2001 Cytoplasmic retention of HIV-1 regulatory protein Vpr by protein-protein interaction with a novel human cytoplasmic protein VprBP. Gene 263:131–140. doi:10.1016/S0378-1119(00)00583-7. 11223251

[11] Lv L, Wang Q, Xu Y, Tsao LC, Nakagawa T, Guo H, Su L, Xiong Y 2018 Vpr targets TET2 for degradation by CRL4(VprBP) E3 ligase to sustain IL-6 expression and enhance HIV-1 replication. Mol Cell 70:961–970 e5. doi:10.1016/j.molcel.2018.05.007. 29883611 PMC6071318

[12] Wu Y, Zhou X, Barnes CO, DeLucia M, Cohen AE, Gronenborn AM, Ahn J, Calero G 2016 The DDB1-DCAF1-Vpr-UNG2 crystal structure reveals how HIV-1 Vpr steers human UNG2 toward destruction. Nat Struct Mol Biol 23:933–940. doi:10.1038/nsmb.3284. 27571178 PMC5385928

[13] Zhou X, DeLucia M, Hao C, Hrecka K, Monnie C, Skowronski J, Ahn J 2017 HIV-1 Vpr protein directly loads helicase-like transcription factor (HLTF) onto the CRL4-DCAF1 E3 ubiquitin ligase. J Biol Chem 292:21117–21127. doi:10.1074/jbc.M117.798801. 29079575 PMC5743084

[14] Bouhamdan M, Benichou S, Rey F, Navarro JM, Agostini I, Spire B, Camonis J, Slupphaug G, Vigne R, Benarous R, Sire J 1996 Human immunodeficiency virus type 1 Vpr protein binds to the uracil DNA glycosylase DNA repair enzyme. J Virol 70:697–704. 8551605 10.1128/jvi.70.2.697-704.1996 PMC189869

[15] Laguette N, Bregnard C, Hue P, Basbous J, Yatim A, Larroque M, Kirchhoff F, Constantinou A, Sobhian B, Benkirane M 2014 Premature activation of the SLX4 complex by Vpr promotes G2/M arrest and escape from innate immune sensing. Cell 156:134–145. doi:10.1016/j.cell.2013.12.011. 24412650

[16] Hrecka K, Hao C, Shun MC, Kaur S, Swanson SK, Florens L, Washburn MP, Skowronski J 2016 HIV-1 and HIV-2 exhibit divergent interactions with HLTF and UNG2 DNA repair proteins. Proc Natl Acad Sci U S A 113:E3921–E3930. doi:10.1073/pnas.1605023113. 27335459 PMC4941427

[17] Lahouassa H, Blondot ML, Chauveau L, Chougui G, Morel M, Leduc M, Guillonneau F, Ramirez BC, Schwartz O, Margottin-Goguet F 2016 HIV-1 Vpr degrades the HLTF DNA translocase in T cells and macrophages. Proc Natl Acad Sci U S A 113:5311–5316. doi:10.1073/pnas.1600485113. 27114546 PMC4868422

[18] Kafer GR, Li X, Horii T, Suetake I, Tajima S, Hatada I, Carlton PM 2016 5-Hydroxymethylcytosine marks sites of DNA damage and promotes genome stability. Cell Rep 14:1283–1292. doi:10.1016/j.celrep.2016.01.035. 26854228

[19] Krokan HE, Bjoras M 2013 Base excision repair. Cold Spring Harb Perspect Biol 5:a012583. doi:10.1101/cshperspect.a012583. 23545420 PMC3683898

[20] Hansen EC, Ransom M, Hesselberth JR, Hosmane NN, Capoferri AA, Bruner KM, Pollack RA, Zhang H, Drummond MB, Siliciano JM, Siliciano R, Stivers JT 2016 Diverse fates of uracilated HIV-1 DNA during infection of myeloid lineage cells. Elife 5 doi:10.7554/eLife.18447. PMC5030084 27644592

[21] Kennedy EM, Daddacha W, Slater R, Gavegnano C, Fromentin E, Schinazi RF, Kim B 2011 Abundant non-canonical dUTP found in primary human macrophages drives its frequent incorporation by HIV-1 reverse transcriptase. J Biol Chem 286:25047–25055. doi:10.1074/jbc.M111.234047. 21454906 PMC3137078

[22] Ciccia A, McDonald N, West SC 2008 Structural and functional relationships of the XPF/MUS81 family of proteins. Annu Rev Biochem 77:259–287. doi:10.1146/annurev.biochem.77.070306.102408. 18518821

[23] Matos J, West SC 2014 Holliday junction resolution: regulation in space and time. DNA Repair (Amst) 19:176–181. doi:10.1016/j.dnarep.2014.03.013. 24767945 PMC4065333

[24] Blastyak A, Hajdu I, Unk I, Haracska L 2010 Role of double-stranded DNA translocase activity of human HLTF in replication of damaged DNA. Mol Cell Biol 30:684–693. doi:10.1128/MCB.00863-09. 19948885 PMC2812231

[25] Guenzel CA, Herate C, Benichou S 2014 HIV-1 Vpr-a still “enigmatic multitasker.” Front Microbiol 5:127. doi:10.3389/fmicb.2014.00127. 24744753 PMC3978352

[26] Vermeire J, Roesch F, Sauter D, Rua R, Hotter D, Van Nuffel A, Vanderstraeten H, Naessens E, Iannucci V, Landi A, Witkowski W, Baeyens A, Kirchhoff F, Verhasselt B 2016 HIV triggers a cGAS-dependent, Vpu- and Vpr-regulated type I interferon response in CD4(+) T cells. Cell Rep 17:413–424. doi:10.1016/j.celrep.2016.09.023. 27705790

[27] Roshal M, Kim B, Zhu Y, Nghiem P, Planelles V 2003 Activation of the ATR-mediated DNA damage response by the HIV-1 viral protein R. J Biol Chem 278:25879–25886. doi:10.1074/jbc.M303948200. 12738771

[28] DeHart JL, Zimmerman ES, Ardon O, Monteiro-Filho CMR, Argañaraz ER, Planelles V 2007 HIV-1 Vpr activates the G2 checkpoint through manipulation of the ubiquitin proteasome system. Virol J 4:57. doi:10.1186/1743-422X-4-57. 17559673 PMC1904188

[29] Le Rouzic E, Belaidouni N, Estrabaud E, Morel M, Rain JC, Transy C, Margottin-Goguet F 2007 HIV1 Vpr arrests the cell cycle by recruiting DCAF1/VprBP, a receptor of the Cul4-DDB1 ubiquitin ligase. Cell Cycle 6:182–188. doi:10.4161/cc.6.2.3732. 17314515

[30] Keijzers G, Liu D, Rasmussen LJ 2016 Exonuclease 1 and its versatile roles in DNA repair. Crit Rev Biochem Mol Biol 51:440–451. doi:10.1080/10409238.2016.1215407. 27494243

[31] Ahn J, Vu T, Novince Z, Guerrero-Santoro J, Rapic-Otrin V, Gronenborn AM 2010 HIV-1 Vpr loads uracil DNA glycosylase-2 onto DCAF1, a substrate recognition subunit of a cullin 4A-ring E3 ubiquitin ligase for proteasome-dependent degradation. J Biol Chem 285:37333–37341. doi:10.1074/jbc.M110.133181. 20870715 PMC2988339

[32] Sharp PM, Hahn BH 2010 The evolution of HIV-1 and the origin of AIDS. Philos Trans R Soc Lond B Biol Sci 365:2487–2494. doi:10.1098/rstb.2010.0031. 20643738 PMC2935100

[33] Elia AE, Boardman AP, Wang DC, Huttlin EL, Everley RA, Dephoure N, Zhou C, Koren I, Gygi SP, Elledge SJ 2015 Quantitative proteomic atlas of ubiquitination and acetylation in the DNA damage response. Mol Cell 59:867–881. doi:10.1016/j.molcel.2015.05.006. 26051181 PMC4560960

[34] Tomimatsu N, Mukherjee B, Harris JL, Boffo FL, Hardebeck MC, Potts PR, Khanna KK, Burma S 2017 DNA-damage-induced degradation of EXO1 exonuclease limits DNA end resection to ensure accurate DNA repair. J Biol Chem 292:10779–10790. doi:10.1074/jbc.M116.772475. 28515316 PMC5491765

[35] Belzile JP, Abrahamyan LG, Gerard FC, Rougeau N, Cohen EA 2010 Formation of mobile chromatin-associated nuclear foci containing HIV-1 Vpr and VPRBP is critical for the induction of G2 cell cycle arrest. PLoS Pathog 6:e1001080. doi:10.1371/journal.ppat.1001080. 20824083 PMC2932712

[36] Zimmerman ES, Chen J, Andersen JL, Ardon O, Dehart JL, Blackett J, Choudhary SK, Camerini D, Nghiem P, Planelles V 2004 Human immunodeficiency virus type 1 Vpr-mediated G2 arrest requires Rad17 and Hus1 and induces nuclear BRCA1 and gamma-H2AX focus formation. Mol Cell Biol 24:9286–9294. doi:10.1128/MCB.24.21.9286-9294.2004. 15485898 PMC522272

[37] Zhao H, Piwnica-Worms H 2001 ATR-mediated checkpoint pathways regulate phosphorylation and activation of human Chk1. Mol Cell Biol 21:4129–4139. doi:10.1128/MCB.21.13.4129-4139.2001. 11390642 PMC87074

[38] Liberti SE, Andersen SD, Wang J, May A, Miron S, Perderiset M, Keijzers G, Nielsen FC, Charbonnier JB, Bohr VA, Rasmussen LJ 2011 Bi-directional routing of DNA mismatch repair protein human exonuclease 1 to replication foci and DNA double strand breaks. DNA Repair (Amst) 10:73–86. doi:10.1016/j.dnarep.2010.09.023. 20970388 PMC4586255

[39] Zhou X, DeLucia M, Ahn J 2016 SLX4-SLX1 protein-independent down-regulation of MUS81-EME1 protein by HIV-1 viral protein R (Vpr). J Biol Chem 291:16936–16947. doi:10.1074/jbc.M116.721183. 27354282 PMC5016100

[40] Nimonkar AV, Genschel J, Kinoshita E, Polaczek P, Campbell JL, Wyman C, Modrich P, Kowalczykowski SC 2011 BLM-DNA2-RPA-MRN and EXO1-BLM-RPA-MRN constitute two DNA end resection machineries for human DNA break repair. Genes Dev 25:350–362. doi:10.1101/gad.2003811. 21325134 PMC3042158

[41] Cohen EA, Dehni G, Sodroski JG, Haseltine WA 1990 Human immunodeficiency virus vpr product is a virion-associated regulatory protein. J Virol 64:3097–3099. 2139896 10.1128/jvi.64.6.3097-3099.1990 PMC249501

[42] Huber HE, McCoy JM, Seehra JS, Richardson CC 1989 Human immunodeficiency virus 1 reverse transcriptase. Template binding, processivity, strand displacement synthesis, and template switching. J Biol Chem 264:4669–4678. 2466838

[43] Miller MD, Wang B, Bushman FD 1995 Human immunodeficiency virus type 1 preintegration complexes containing discontinuous plus strands are competent to integrate in vitro. J Virol 69:3938–3944. 7745750 10.1128/jvi.69.6.3938-3944.1995 PMC189122

[44] Hrecka K, Hao C, Gierszewska M, Swanson SK, Kesik-Brodacka M, Srivastava S, Florens L, Washburn MP, Skowronski J 2011 Vpx relieves inhibition of HIV-1 infection of macrophages mediated by the SAMHD1 protein. Nature 474:658–661. doi:10.1038/nature10195. 21720370 PMC3179858

[45] Srivastava S, Swanson SK, Manel N, Florens L, Washburn MP, Skowronski J 2008 Lentiviral Vpx accessory factor targets VprBP/DCAF1 substrate adaptor for cullin 4 E3 ubiquitin ligase to enable macrophage infection. PLoS Pathog 4:e1000059. doi:10.1371/journal.ppat.1000059. 18464893 PMC2330158

[46] Goujon C, Moncorge O, Bauby H, Doyle T, Ward CC, Schaller T, Hue S, Barclay WS, Schulz R, Malim MH 2013 Human MX2 is an interferon-induced post-entry inhibitor of HIV-1 infection. Nature 502:559–562. doi:10.1038/nature12542. 24048477 PMC3808269

[47] Foley GE, Lazarus H, Farber S, Uzman BG, Boone BA, McCarthy RE 1965 Continuous culture of human lymphoblasts from peripheral blood of a child with acute leukemia. Cancer 18:522–529. doi:10.1002/1097-0142(196504)18:4<522::AID-CNCR2820180418>3.0.CO;2-J. 14278051

[48] Nara PL, Hatch WC, Dunlop NM, Robey WG, Arthur LO, Gonda MA, Fischinger PJ 1987 Simple, rapid, quantitative, syncytium-forming microassay for the detection of human immunodeficiency virus neutralizing antibody. AIDS Res Hum Retroviruses 3:283–302. doi:10.1089/aid.1987.3.283. 3481271

[49] Thangavel S, Berti M, Levikova M, Pinto C, Gomathinayagam S, Vujanovic M, Zellweger R, Moore H, Lee EH, Hendrickson EA, Cejka P, Stewart S, Lopes M, Vindigni A 2015 DNA2 drives processing and restart of reversed replication forks in human cells. J Cell Physiol 208:545–562. doi:10.1083/jcb.201406100. PMC4347643 25733713

[50] Averbeck NB, Ringel O, Herrlitz M, Jakob B, Durante M, Taucher-Scholz G 2014 DNA end resection is needed for the repair of complex lesions in G1-phase human cells. Cell Cycle 13:2509–2516. doi:10.4161/15384101.2015.941743. 25486192 PMC4615131

[51] D'Angiolella V, Donato V, Forrester FM, Jeong YT, Pellacani C, Kudo Y, Saraf A, Florens L, Washburn MP, Pagano M 2012 Cyclin F-mediated degradation of ribonucleotide reductase M2 controls genome integrity and DNA repair. Cell 149:1023–1034. doi:10.1016/j.cell.2012.03.043. 22632967 PMC3616325

[52] Fellmann C, Hoffmann T, Sridhar V, Hopfgartner B, Muhar M, Roth M, Lai DY, Barbosa IA, Kwon JS, Guan Y, Sinha N, Zuber J 2013 An optimized microRNA backbone for effective single-copy RNAi. Cell Rep 5:1704–1713. doi:10.1016/j.celrep.2013.11.020. 24332856

[53] Janardhan A, Swigut T, Hill B, Myers MP, Skowronski J 2004 HIV-1 Nef binds the DOCK2-ELMO1 complex to activate rac and inhibit lymphocyte chemotaxis. PLoS Biol 2:E6. doi:10.1371/journal.pbio.0020006. 14737186 PMC314466

[54] Ahn J, Hao C, Yan J, DeLucia M, Mehrens J, Wang C, Gronenborn AM, Skowronski J 2012 HIV/simian immunodeficiency virus (SIV) accessory virulence factor Vpx loads the host cell restriction factor SAMHD1 onto the E3 ubiquitin ligase complex CRL4DCAF1. J Biol Chem 287:12550–12558. doi:10.1074/jbc.M112.340711. 22362772 PMC3321004

[55] Wilson IA, Niman HL, Houghten RA, Cherenson AR, Connolly ML, Lerner RA 1984 The structure of an antigenic determinant in a protein. Cell 37:767–778. doi:10.1016/0092-8674(84)90412-4. 6204768

[56] Chesebro B, Wehrly K, Nishio J, Perryman S 1992 Macrophage-tropic human immunodeficiency virus isolates from different patients exhibit unusual V3 envelope sequence homogeneity in comparison with T-cell-tropic isolates: definition of critical amino acids involved in cell tropism. J Virol 66:6547–6554. 1404602 10.1128/jvi.66.11.6547-6554.1992 PMC240149

