# Supplementary figures and images for: HIV-1 Vpr Reprograms CLR4DCAF1 E3 Ubiquitin Ligase to Antagonize Exonuclease 1-Mediated Restriction of HIV-1 Infection
Source: mBio. 2018 Oct 23;9(5):e01732-18. doi: 10.1128/mBio.01732-18 (PMC6199497; doi:10.1128/mBio.01732-18)

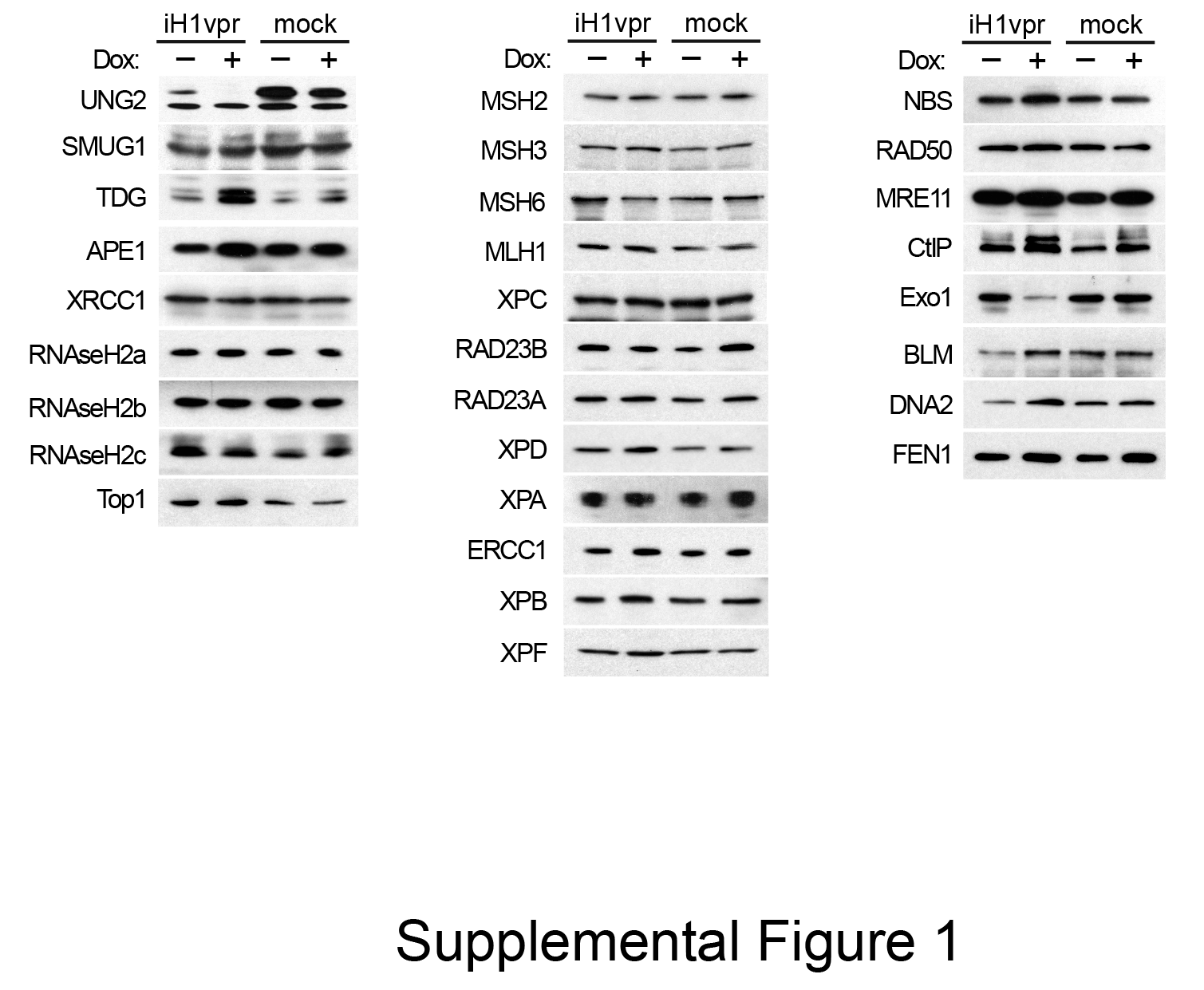

Supplement: FIG S1 [file mbo005184128sf1.tif]

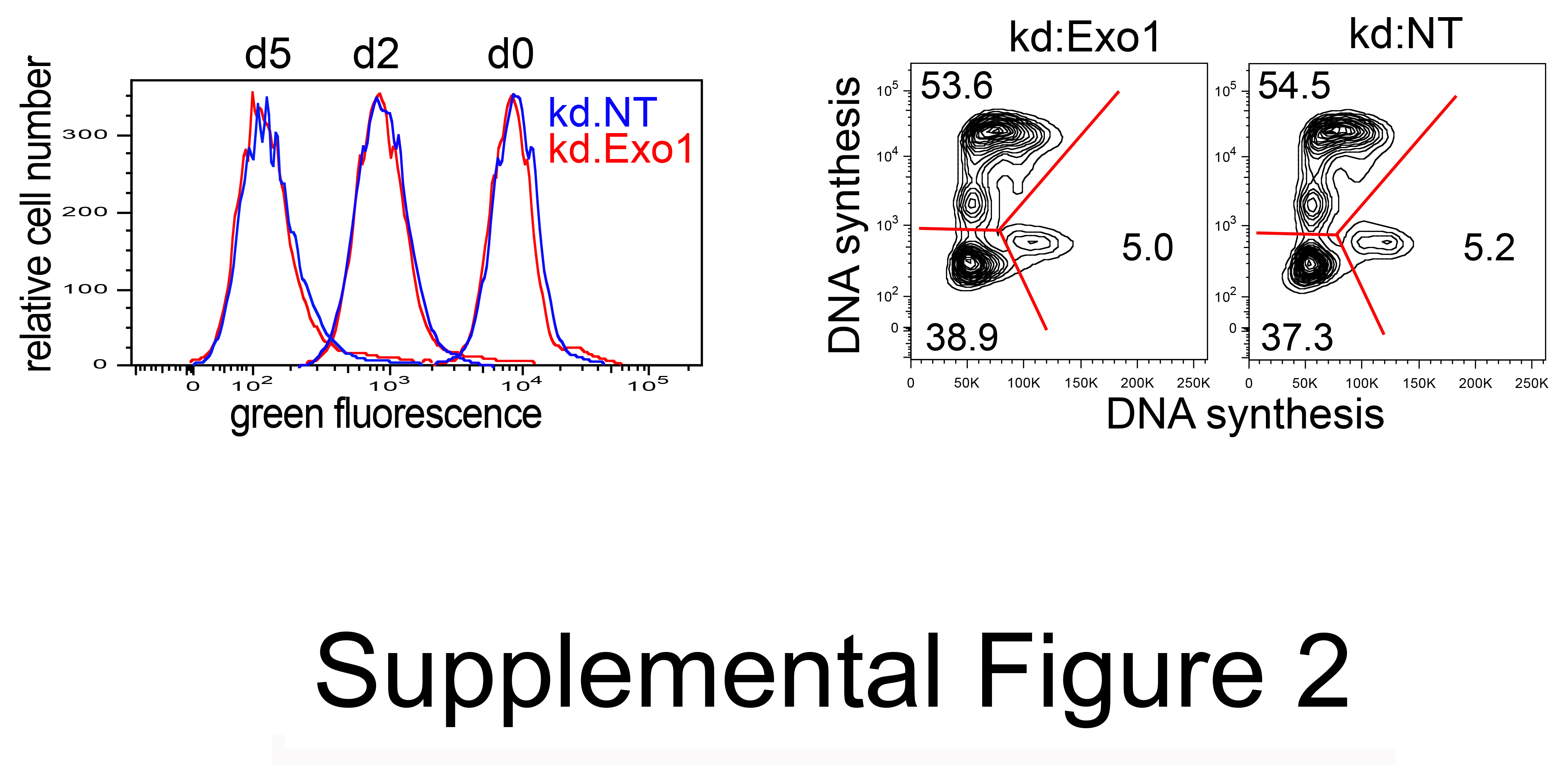

Supplement: FIG S2 [file mbo005184128sf2.tif]
